# Targeted Deletion of *Kcne2* Impairs HCN Channel Function in Mouse Thalamocortical Circuits

**DOI:** 10.1371/journal.pone.0042756

**Published:** 2012-08-03

**Authors:** Shui-Wang Ying, Vikram A. Kanda, Zhaoyang Hu, Kerry Purtell, Elizabeth C. King, Geoffrey W. Abbott, Peter A. Goldstein

**Affiliations:** 1 Department of Anesthesiology, Weill Cornell Medical College, New York, New York, United States of America; 2 Department of Pharmacology, Weill Cornell Medical College, New York, New York, United States of America; 3 Departments of Pharmacology, and Physiology and Biophysics, University of California Irvine, Irvine, California, United States of America; Sackler Medical School, Tel Aviv University, Israel

## Abstract

**Background:**

Hyperpolarization-activated, cyclic nucleotide-gated (HCN) channels generate the pacemaking current, I_h_, which regulates neuronal excitability, burst firing activity, rhythmogenesis, and synaptic integration. The physiological consequence of HCN activation depends on regulation of channel gating by endogenous modulators and stabilization of the channel complex formed by principal and ancillary subunits. KCNE2 is a voltage-gated potassium channel ancillary subunit that also regulates heterologously expressed HCN channels; whether KCNE2 regulates neuronal HCN channel function is unknown.

**Methodology/Principal Findings:**

We investigated the effects of *Kcne2* gene deletion on I_h_ properties and excitability in ventrobasal (VB) and cortical layer 6 pyramidal neurons using brain slices prepared from *Kcne2* ^+/+^ and *Kcne2* ^−/−^ mice. *Kcne2* deletion shifted the voltage-dependence of I_h_ activation to more hyperpolarized potentials, slowed gating kinetics, and decreased I_h_ density. *Kcne2* deletion was associated with a reduction in whole-brain expression of both HCN1 and HCN2 (but not HCN4), although co-immunoprecipitation from whole-brain lysates failed to detect interaction of KCNE2 with HCN1 or 2. *Kcne2* deletion also increased input resistance and temporal summation of subthreshold voltage responses; this increased intrinsic excitability enhanced burst firing in response to 4-aminopyridine. Burst duration increased in corticothalamic, but not thalamocortical, neurons, suggesting enhanced cortical excitatory input to the thalamus; such augmented excitability did not result from changes in glutamate release machinery since miniature EPSC frequency was unaltered in *Kcne2* ^−/−^ neurons.

**Conclusions/Significance:**

Loss of KCNE2 leads to downregulation of HCN channel function associated with increased excitability in neurons in the cortico-thalamo-cortical loop. Such findings further our understanding of the normal physiology of brain circuitry critically involved in cognition and have implications for our understanding of various disorders of consciousness.

## Introduction

The pacemaker current I_h_, which is generated by hyperpolarization-activated, cyclic nucleotide-gated (HCN) channels, regulates intrinsic excitability, synaptic integration and rhythmic oscillatory activity [1]–[3]. There are four *Hcn* genes, each coding for a distinct isoform (HCN1-4) (reviewed by Biel *et al.* 2009 [3]), which are variably distributed in the brain [4]. Although permeable to both Na^+^ and K^+^, HCN channels are members of the voltage-gated potassium channel superfamily. HCN channels are not inhibited by the inwardly rectifying K^+^ channel blockers Ba^2+^ or tetraethlylammonium, nor the voltage-gated K^+^ channel blocker 4-aminopyrindine, although they are inhibited by several different organic blockers, including ZD7288 [1], [3].

KCNE2, originally named MinK-related protein 1 (MiRP1), is a single transmembrane-spanning protein that acts as an ancillary (β) subunit for a number of potassium channel pore-forming α subunits, regulating channel conductance, voltage dependence, gating kinetics, trafficking and pharmacology [5]–[9] (for review see [10]). Studies using heterologous or over-expression systems have shown that co-expression of KCNE2 with HCN1, 2 or 4 significantly alters the amplitude and kinetics of I_h_ with variable effects on voltage-dependent gating [11]–[14]. KCNE2 also increases HCN1, HCN2, and HCN4 single channel conductance, further suggesting a direct interaction [14]. Despite these observations, however, the impact of KCNE2 expression on brain HCN channel function is unknown.

*Kcne2* mRNA is present in many brain regions [15] where HCN isoforms are strongly expressed [16]–[18], raising the possibility that KCNE2 could directly influence the function of HCN channels in central neurons. KCNE2 is also highly expressed in the apical membrane of the choroid plexus epithelium, where it influences cerebrospinal fluid composition by regulating KCNQ1 and Kv1.3 K^+^ channel α subunits [19], potentially also indirectly influencing neuronal excitability. Thalamic neurons express HCN2 and HCN4, with HCN2 being the major functional isoform [20], [21] while cortical pyramidal neurons strongly express HCN1 [4].

Dysregulation of HCN channel function is strongly implicated in various experimental seizure models [22], [23] as well as in human epilepsy [24]. Changes in cellular excitability within corticothalamic circuits can result in seizure activity [25]–[27]. The corticothalamocortical circuit consists of reciprocal connections between the cortex and thalamus such that thalamic VB neurons project to layer 4 and 6 of the somatosensory cortex [28], and layer 6 pyramidal neurons in turn send axons to thalamic neurons, including those in VB [28], [29]. Thus, the thalamus and cortex are ideal regions to study the effects of KCNE2 on HCN channel function.

Here, using *Kcne2* ^+/+^ and *Kcne2* ^−/−^ mice, we have discovered that targeted *Kcne2* deletion alters I_h_ properties and neuronal excitability in VB and somatosensory cortex layer 6 neurons and reduces HCN1 and HCN2 protein expression in the brain. Preliminary results have been previously reported [30].

## Methods

### Ethics statement

All experiments were performed following approval by, and in accordance with, Weill Cornell Medical College, University of California, and US federal guidelines.

### Generation of Kcne2^−/−^ mice

*Kcne2* ^+/+^ and *Kcne2* ^−/−^ C57BL/6 mice used in this study were generated by breeding *Kcne2* ^+/−^ pairs and genotyped as described previously [31], [32].

### Electrophysiology

A total of 78 mice of either sex (P60–96) were used for preparation of brain slices. Electrophysiological experiments in either current or voltage clamp configuration were performed as previously described [33], [34], and methods were slightly modified for this study. Briefly, thalamocortical slices (200–300 µm) were prepared using ice-cold slicing solution containing (in mM): 2 KCl, 26 NaHCO_3_, 1.25 NaH_2_PO_4_, 240 sucrose, 12 glucose, 2 MgSO_4_, 1 MgCl_2_, and 1 CaCl_2_. Whole-cell patch-clamp recordings were made from visually identified neurons in thalamic ventrobasal (VB) complex and somatosensory cortex layer 6. Slices were perfused with carbogenated normal artificial cerebrospinal fluid (ACSF), which contained (in mM): 126 NaCl, 26 NaHCO_3_, 3.6 KCl, 1.2 NaH_2_PO_4_, 1.2 MgCl_2_, 2 CaCl_2_, and 17 glucose. To isolate I_h_ currents in voltage-clamp recordings, an “I_h_ isolation solution” was used with the following compounds added to the ACSF (in mM): 0.001 tetrodotoxin (TTX, from Alomone Labs, Jerusalem, Israel), 2 4-aminopyridine (4-AP), 1 BaCl_2_ and 0.1 NiCl_2_; in some cases (n = 10), 0.02 6-cyano-7-nitroquinoxaline-2,3-dione (CNQX: Tocris Bioscience, Ellisville, MO) and 0.04 DL-2-amino-5-phosphonopentanoic acid (AP5; Tocris) were included. For recordings of I_h_ and firing activity, the intracellular solution contained (in mM): 135 K^+^-gluconate, 5 NaCl, 10 HEPES, 0.5 EGTA, 3 K_2_-ATP, 0.2 Na-GTP, and 10 Na_2_-phosphocreatine, pH adjusted to 7.3 with KOH. For recordings of excitatory postsynaptic currents (EPSCs), the intracellular solution contained (in mM): 130 CH_3_SO_3_Cs, 10 CH_3_SO_3_Na, 5 NaCl, 1 CaCl_2_, 10 EGTA, 2 Mg_2_-ATP, 0.3 Na-GTP, and 10 HEPES, pH adjusted to 7.2 with CsOH. To obtain miniature EPSCs (mEPSCs), bicuculline (20 µM) and TTX (1 µM) were included in normal ACSF; neurons were clamped at −80 mV. CNQX (20 µM) and AP5 (40 µM) were used to identify EPSCs. All chemicals and drugs were purchased from Sigma unless otherwise noted.

Access resistance and capacitance were compensated after a whole-cell configuration was established, and were monitored throughout recordings; data were discarded if either of the two parameters changed by >20% of the original values. Liquid junction potentials were calculated and corrected off-line [35]. For recordings of I_h_, neurons were voltage-clamped at −50 mV; 10- and 5-s hyperpolarizing voltage steps respectively were applied to VB and cortical neurons from −50 to −120 mV (10 mV/step).

### Protein biochemistry

Co-immunoprecipitations (co-IPs) and associated western blots were performed as previously described [36] using brains from *Kcne2* ^+/+^ and *Kcne2* ^−/−^ mice (∼P 90). For western blot analysis of whole-brain HCN protein expression (independent of the co-IP experiments), brain tissue was obtained from *Kcne2* ^+/+^ and *Kcne2* ^−/−^ mice (∼P 90), homogenized in microcentrifuge tubes using motorized disposable pestles, then solubilized in PBS containing 20% (w/v) sodium dodecyl sulfate (SDS). Lysates were heated to 42°C for 30 minutes with multiple vortex mixing steps, centrifuged at 12,000 g for 15 minutes, and the supernatant retained. Supernatants (40 µg protein/lane) were size-fractionated on 8–12% Bis-Tris gels (Invitrogen, Grand Island, NY) with MES running buffer (HCN2, GAPDH; glyceraldehyde-3-phosphate dehydrogenase) or 3–8% Tris-Acetate gels (Invitrogen) with Tris-Acetate SDS running buffer (Invitrogen) (HCN1, GAPDH, Kv2.1, KCC1), transferred onto polyvinylidene difluoride (PVDF) membranes, and probed with mouse monoclonal antibodies against HCN1, HCN2 or HCN4 (UC Davis/NIH NeuroMab Facility), and GAPDH loading control (Sigma), or rabbit polyclonal antibodies raised against Kv2.1 (Sigma) or KCC1 (Chemicon/Millipore, Temecula, CA). Horseradish peroxidase (HRP)-conjugated goat anti-mouse or anti-rabbit immunoglobulin G (IgG) secondary antibody (Bio-Rad Labs, Hercules, CA) was used for visualization with chemiluminescence (ECL Plus; Amersham Biosciences, Piscataway, NJ, USA). Band intensities were compared directly from PVDF membrane chemiluminescence using the GBox imager (Syngene, Frederick, MD) after normalization for total protein concentration using the Bicinchoninic Acid (BCA) assay.

### Data Analysis

Data processing and analysis including the construction of I_h_ activation curves, fit of time constants, and measurements of temporal summation, burst and tonic spike firing were performed using MiniAnalysis (Synaptosoft, Decatur, GA) or Clampfit 10 (Molecular Devices, Foster City, CA) as previously described [34]. Steady-state activation curves of I_h_ currents were determined from the tail current (for VB neurons, tail currents were analyzed upon returning to −50 mV while in cortical neurons, the tail current was measured at −60 mV); normalized tail current values were plotted as a function of the voltage steps, and were fitted with the Boltzmann function. Activation time constants were determined by fitting 4-s and 1-s segments of the current trace using a double exponential function [18], while tail current traces were fitted with a single exponential to obtain deactivation kinetics. Data are presented as mean ± SEM; statistical significance was determined using Student's *t* test or one-way ANOVA with pairwise comparisons, as appropriate.

## Results

### Kcne2 deletion impairs HCN channel function in VB neurons

**Figure 1:**
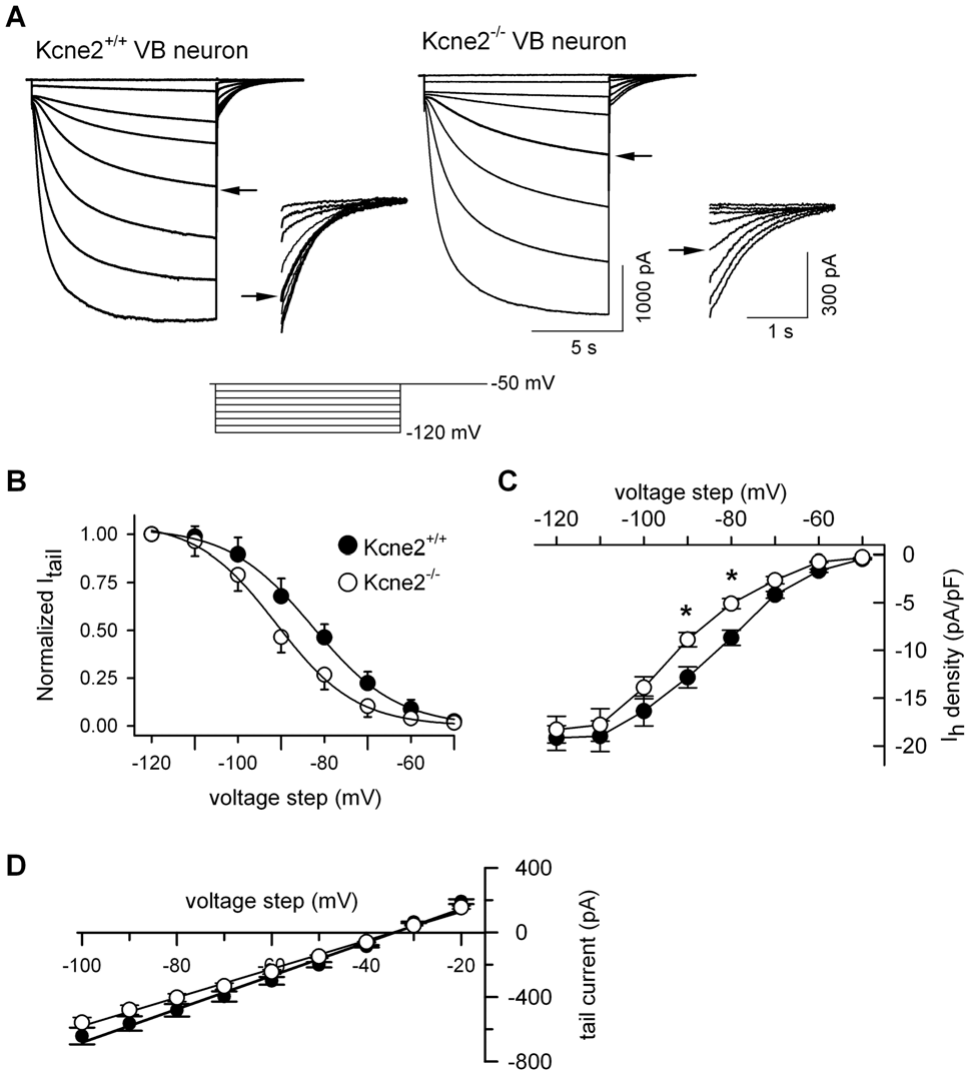
Deletion of *Kcne*2 results in a hyperpolarizing shift in HCN channel activation in VB neurons. ***A***, Representative families of current traces recoded in VB neurons from *Kcne2^+/+^* and *Kcne2^−/−^* mice; the voltage protocol is shown below. The tail currents from the same neurons are shown on an expanded time scale for comparison. Arrow indicates the trace elicited at −90 mV. ***B***, Normalized tail currents (I_tail_) are plotted as a function of voltage steps and are fit with the Boltzmann equation. V_1/2_ values (in mV) were significantly shifted by the deletion to more hyperpolarized potentials. ***C***, Comparison of I_h_ density in the two genotypes. *, P<0.01, one-way ANOVA, *vs. Kcne2^+/+^*. ***D***, Deletion of *Kcne*2 did not alter I_h_ reversal potential.

VB neurons predominantly express HCN2 (and to a lesser extent HCN4) subunits in the soma and generate a large I_h_ current which slowly activates [20], [21], making the VB an excellent region in which to gain insights into whether KCNE2 influences native HCN function. We therefore compared properties of I_h_ currents recorded from VB neurons in brain slices prepared from *Kcne2* ^+/+^ and *Kcne2* ^−/−^ mice. Families of I_h_ current traces were elicited by a series of 10 s hyperpolarizing voltage steps (Fig. 1A). Other voltage-gated ion channels (Na^+^, Ca^2+^ and K^+^) were blocked by concomitant application of tetrodotoxin, Ni^2+^, Ba^2+^ and 4-aminopyridine (see Methods). To determine the dependence of HCN channel activation on voltage, we measured tail current amplitudes at −50 mV following application of hyperpolarizing voltage steps. Tail currents were normalized to maximum amplitude and normalized values were fitted with the Boltzmann function (Fig. 1B) to construct activation curves. Group data demonstrate that there was a significant difference in the mid-point voltage of steady-state activation (V_1/2_ in mV): −84.3±0.7 for *Kcne2* ^+/+^ and −91.8±0.9 for *Kcne2* ^−/−^ (P<0.001, t-test, n = 37/genotype); slope (mV) was not altered by the deletion.

All VB neurons recorded demonstrated a robust I_h_ (up to 5,500 pA) in both genotypes. Currents were normalized to cell capacitance to obtain I_h_ density (pA/pF, Fig. 1C). Although the maximum I_h_ density at −120 mV was not different between *Kcne2* ^+/+^ and *Kcne2* ^−/−^ mice, *Kcne2* deletion reduced I_h_ density in *Kcne2* ^−/−^ neurons at physiological potentials (−80 and −90 mV, Fig. 1C) by virtue of the hyperpolarizing shift in the voltage dependence of activation (Fig. 1 B). Instantaneous I_h_ currents were not significantly changed by *Kcne2* deletion (not shown).

To examine whether *Kcne2* deletion altered the ion selectivity of VB HCN channels, I_h_ reversal potential was measured as previously described [33] from tail currents elicited by a series of voltage steps and plotted as a function of pre-pulse voltage. I_h_ reversal potential, and therefore HCN ion selectivity, was not significantly changed by *Kcne2* deletion (*Kcne2* ^+/+^, −34.5±1.2 mV; *Kcne2* ^−/−^, −33.4±0.8 mV, n = 3 cells/genotype) (Fig. 1D).

**Figure 2:**
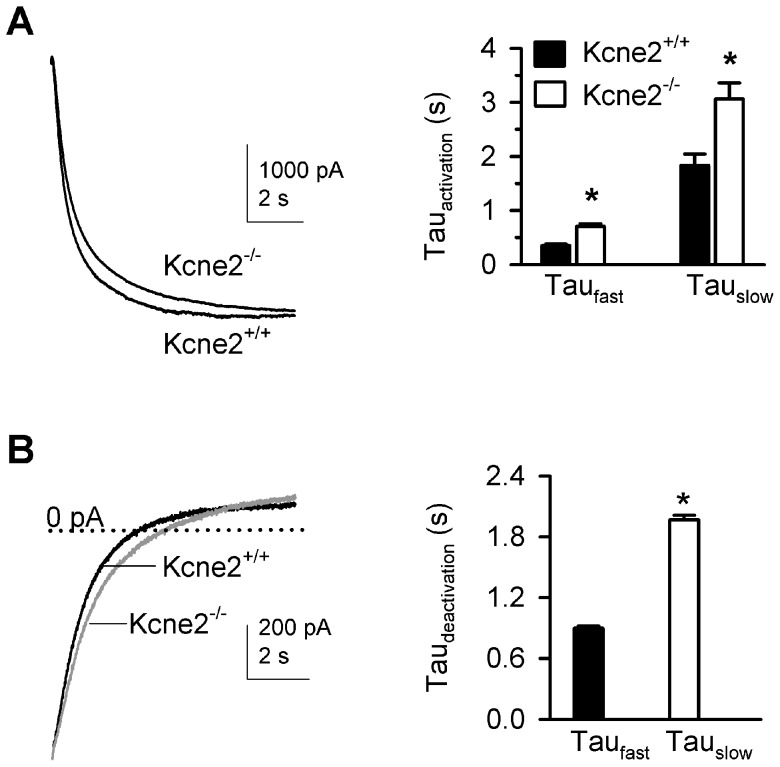
Deletion of *Kcne2* slows the activation and deactivation of HCN channels in VB neurons. ***A***, Overlay of current traces showing the effect of *Kcne2* deletion on the activation time course of I_h_ at −120 mV. The activation time constant (Tau_activation_) is determined by a two-exponential function, yielding fast (Tau_fast_) and slow (Tau_slow_) components. Bar graph comparing the activation time constants. *, P<0.01, t-test, n = 26/genotype. ***B***, Overlay of current traces showing the differences in the deactivation time of I_h_; the *Kcne2* ^−/−^ current trace (gray) is scaled to that of the *Kcne2* ^+/+^ trace (black) so as to better observe the kinetic differences in the currents. The dotted line indicates 0 pA. Bar graph summarizing the effect of *Kcne2* deletion on the deactivation time constant (Tau_deactivation_). * P<0.05, t-test, n = 26/genotype.

We also analyzed the kinetics of channel activation by using fits of traces elicited at −120 mV with two exponential components. The fast time component (Tau_fast_) was 0.35±0.026 s for *Kcne2* ^+/+^ and 0.707±0.05 s for *Kcne2* ^−/−^, and the slow time component (Tau_slow_) was 1.84±0.21 s for *Kcne2* ^+/+^ and 3.07±0.29 for *Kcne2* ^−/−^ (Fig. 2A). Tail currents measured at −50 mV following a voltage step to −120 mV were fitted with a single exponential to quantify deactivation kinetics. *Kcne2* deletion also increased the deactivation time constant (0.898±0.02 s *vs.* 1.97±0.045 s, Fig. 2B). Thus, *Kcne2* deletion slowed the kinetics of both activation and deactivation, doubling the time constants for both processes.

**Figure 3:**
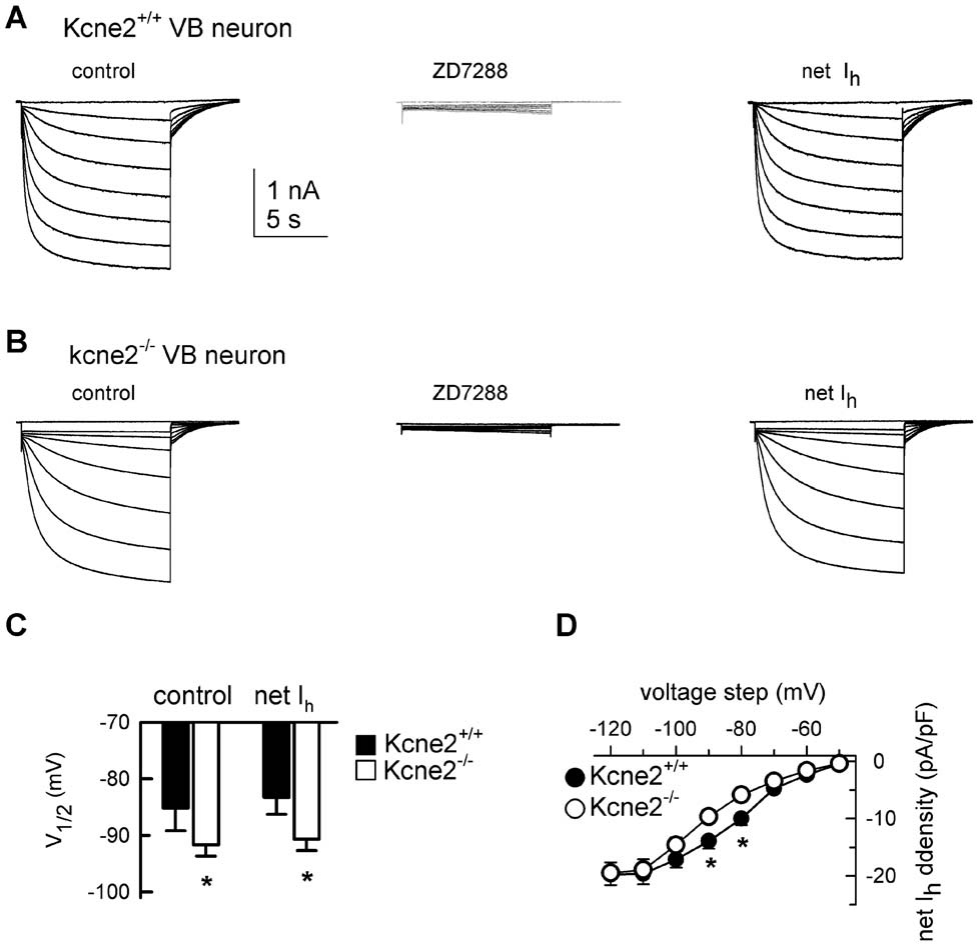
*Kcne2* deletion does not alter I_h_ sensitivity to the HCN channel blocker ZD7288 in VB neurons. ***A*** and ***B***, Examplar families of current traces in the absence (control) and presence of ZD7288 (50 µM). Data were obtained using the same protocol as in Fig. 1A. Net I_h_ = control – ZD7288. ***C***, Bar graph summarizing the effect of *Kcne2* deletion on V_1/2_. *, P<0.001, t-test, n = 27 for control (no ZD7288) data and n = 16 for data with ZD7288. ***D***, Comparison of net I_h_ density as a function of voltage. *, P<0.05, one-way ANOVA, *vs. Kcne2^+/+^*; the number of neurons is the same as in *C*.

To support the hypothesis that the altered VB currents in *Kcne2* ^−/−^ mice arose from shifts in I_h_ rather than other currents, we compared the functional attributes of VB currents pharmacologically isolated using the I_h_ blocker ZD7288 in *Kcne2* ^+/+^ and *Kcne2* ^−/−^ mice. Thus, “net” I_h_, was calculated by subtracting current traces obtained in the presence of ZD7288 (50 µM) from “control” traces (no ZD7288). Comparison of control I_h_ with net I_h_ demonstrated no significant difference in HCN channel properties between these two groups within a given genotype, yet the genotype-dependent differences we observed using control I_h_, *i.e*., a shift in voltage dependence (Fig. 1B) were recapitulated with net I_h_, Thus, V_1/2_ values for control and net I_h_, respectively, were (in mV) −85.2±2 *vs.* −83.3±2.6 for *Kcne2* ^+/+^ and −91.7±2 vs. −90.7±1.8 for *Kcne2* ^−/−^ (Fig. 3C). The relationship of current-voltage curves was also the same for control versus net I_h_ within a given genotype (Fig. 3D). The data support the hypothesis that changes in I_h_ properties arising from *Kcne2* deletion result from altered HCN channel function.

### Kcne2 deletion markedly down-regulates I_h_ in cortical pyramidal neurons

**Figure 4:**
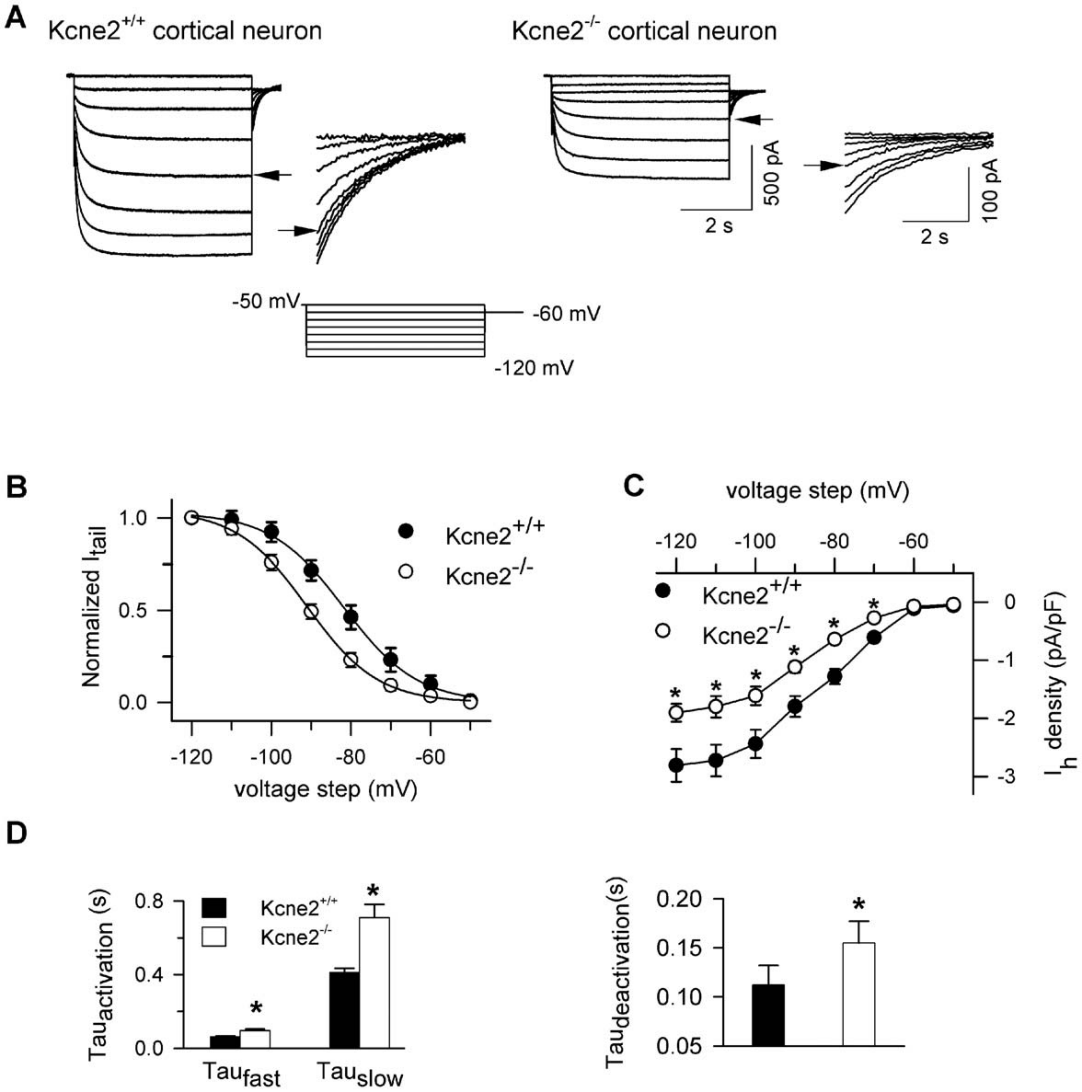
Loss of *Kcne*2 markedly down-regulates HCN channel function in layer 6 pyramidal neurons. ***A***, Families of I_h_ current traces were recorded in layer 6 neurons from both genotypes using the voltage protocol shown. Tail currents are shown on an expanded time scale for better comparison. Arrow indicates the trace elicited at −90 mV. ***B***, Steady-state activation curves show that loss of *Kcne*2 resulted in a significant negative shift in voltage dependence (V_1/2_ in mV: −81.2±0.6 for *Kcne2^+/+^ vs.* −91.4±0.7 for *Kcne2^−/−^*, t-test, n = 15/genotype), with no apparent change in slope. ***C***, Comparison of I_h_ density, *, P<0.05, one way ANOVA, vs. *Kcne2^+/+^*. ***D***, Bar graphs summarizing the effects of *Kcne2* deletion on the kinetics of HCN channel activation and deactivation. *, P<0.01.

*Kcne*2 mRNA is detected at relatively high density in cortical dendritic regions [15], where HCN1 is also predominantly expressed [4], [37]. We therefore examined the potential influence of *Kcne2* deletion on I_h_ properties in cortical layer 6 pyramidal (corticothalamic) neurons. Families of I_h_ current traces were obtained (Fig. 4A) using the same I_h_ isolation solution as for VB neurons. Analysis of steady-state activation curves (Fig. 4B) revealed that the deletion markedly shifted voltage dependence to more hyperpolarized potentials by 10.2±0.8 mV, with the V_1/2_ (in mV) being −81.2±0.6 for *Kcne2* ^+/+^ pyramidal neurons and −91.4±0.7 for *Kcne2* ^−/−^ neurons (P<0.001, t-test, n = 10/genotype). There was no significant change in slope (9.2±0.6 *vs.* 8.9±0.5 mV, respectively). Unlike the scenario in VB, I_h_ current density (pA/pF) measured in these pyramidal neurons was small, and the maximal density detected at −120 mV was 2.8±0.26 pA/pF for *Kcne2* ^+/+^ and 1.9±0.15 pA/pF for *Kcne2* ^−/−^. The low I_h_ density in either genotype is consistent with uneven distribution of HCN subunits along the somatodendritic axis, with very low density over the somata and extremely high density in distal dendrites [4], [37], [38].

Unlike VB neurons, *Kcne2* ^−/−^ pyramidal neurons displayed a significant decrease in I_h_ current density across the −70 to −120 mV range (Fig. 4C). While activation time constants for I_h_ in pyramidal cells were fast in both genotypes compared to those in VB neurons, *Kcne2* deletion again increased time constants in pyramidal neurons (*Kcne2* ^+/+^: Tau_fast_, 0.06±0.004 s, Tau_slow_, 0.41±0.02 s; *Kcne2* ^−/−^: Tau_fast_, 0.097±0.007, Tau_slow_, 0.71±0.07). The kinetics of both activation and deactivation for I_h_ were slowed in *Kcne2* ^−/−^ pyramidal neurons (Fig. 4D). The kinetic data strongly suggest that I_h_ recorded from cortical pyramidal neurons is primarily generated by the HCN1 isoform [39]–[42] and slowed by *Kcne2* deletion.

### Kcne2 deletion enhances excitability and susceptibility to 4-AP in VB neurons

**Figure 5:**
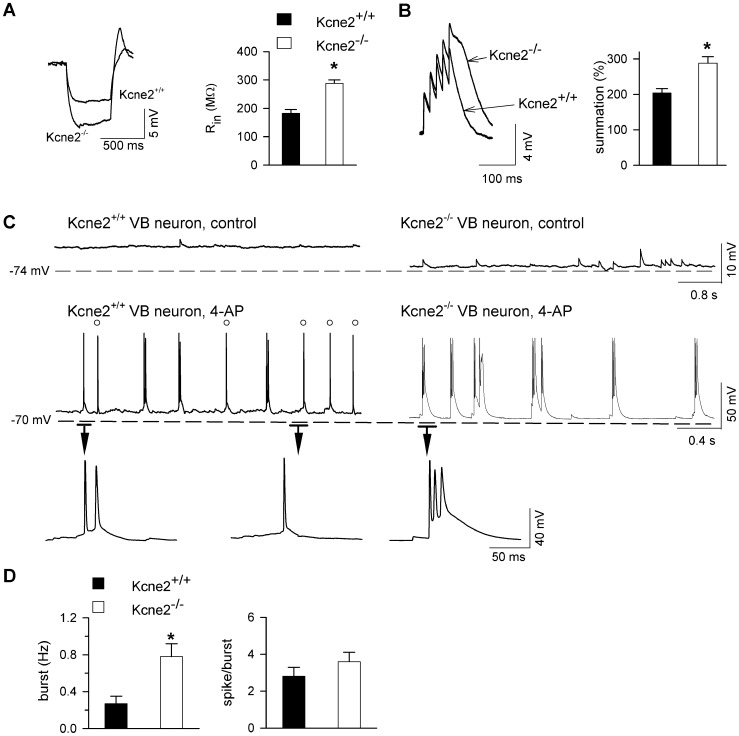
Deletion of *Kcne*2 increases sensitivity of VB neurons to the convulsant 4-AP. ***A***, Comparison of input resistance (R_in_) between genotypes. Voltage traces were elicited by intracellular injection of a hyperpolarizing current pulse (−30 pA, 500 ms) at holding potential of −70 mV. *, P<0.01. ***B***, Comparison of intrinsic temporal summation of the voltage response in representative VB neurons from each genotype. Voltage traces were elicited by intracellular injection of an EPSC-shared train (5 pulses, 200 pA, 33 Hz). *, P<0.01, t-test. ***C***, Voltage traces showing spontaneous activity in the absence (control) and presence of 4-aminopyridine (4-AP). The first burst from each genotype is expanded for better view of fast spikes. Circles indicate single fast action potentials; a single spike in the Kcne2^+/+^ neuron is shown for comparison. ***D***. Bar graph summarizing burst firing data. *, P<0.05, t-test, n = 12/genotype.

Down-regulation of HCN channel function by *Kcne2* deletion would be predicted to alter intrinsic and synaptic excitability, and this possibility was investigated here. The resting membrane potential (RMP) was −68.6±0.7 mV for *Kcne2* ^+/+^ VB neurons compared to −72.1±0.8 mV for *Kcne2* ^−/−^ VB neurons (n = 20). A small voltage response was elicited for measurement of input resistance by intracellular injection of a hyperpolarizing current pulse (−30 pA, 500 ms); *Kcne2* deletion significantly increased input resistance from 182±14 to 288±12 MΩ (n = 12/genotype; Fig. 5A). Intrinsic temporal summation of subthreshold voltage response was evoked by intracellular injection of an EPSC-shaped current train [35], [43]. As shown in Fig. 5B, temporal summation (%) was 204±12 in *Kcne2* ^+/+^ neurons, and was significantly increased to 288±18 (P<0.01, n = 8/genotype) in neurons from *Kcne2* ^−/−^ mice. Thus, *Kcne2* deletion increased intrinsic excitability in VB neurons, raising the question as to whether excitatory synaptic transmission was altered.

Spontaneous EPSPs were evident in both genotypes (Fig. 5C, and see below for additional information) and could be blocked by CNQX and AP5 (not shown). As very few spontaneous bursts were observed in either genotype under control conditions, we used a low concentration of 4-aminopyridine (4-AP; 0.1 mM) to study burst firing and neuronal sensitivity to convulsant challenge. Bath-application of 4-AP induced low-threshold bursts in both genotypes. Fast single action potentials occurred between bursts in *Kcne2* ^+/+^ neurons but few appeared in *Kcne2* ^−/−^ cells. Group data indicate that the burst frequency was significantly higher in *Kcne2* ^−/−^ neurons than that in *Kcne2* ^+/+^ neurons (Fig. 5 D) although no significant change in burst duration or spikes/burst was observed. These data indicate that *Kcne2* deletion increases intrinsic excitability and facilitates low-threshold burst firing in thalamocortical VB neurons.

### Loss of kcne2 produces hypersusceptibility to 4-AP in layer 6 pyramidal neurons

**Figure 6:**
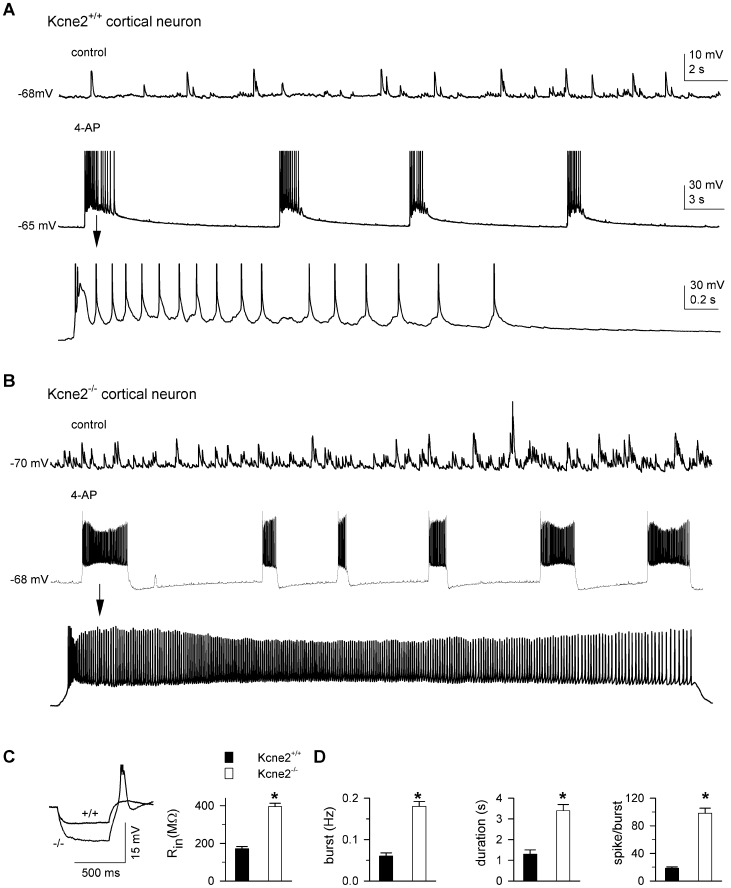
Loss of *Kcne2* results in hypersusceptibility to 4-AP in cortical layer 6 pyramidal neurons of somatosensory cortex. ***A*** and ***B***, Representative voltage traces in the absence (control) and presence of 4-aminopyridine (4-AP) in both genotypes. The first burst (marked by arrow) is shown on an expanded time scale to better view fast spikes. *Kcne2* ^−/−^ pyramidal neurons exhibited prolonged bursts (increased burst duration), as compared to wild-types. ***C***, Voltage responses were elicited to determine input resistance (R_in_) by intracellular injections of a hyperpolarizing current pulse (−30 pA, 500 ms). +/+, *Kcne2^+/+^*; −/−, *Kcne2^−/−^*. Bar graph showing comparison of R_in_ values in the two genotypes. *, P<0.00, t-test. ***D***, Bar graphs summarizing burst firing properties. *, P<0.05, t-test.

Since I_h_ density was significantly reduced across the voltage range in *Kcne2^−/−^* layer 6 pyramidal neurons (Fig. 4C), we also examined whether excitability in these cells was altered. Input resistance significantly increased in *Kcne2* ^−/−^ pyramidal neurons compared to those from *Kcne2* ^+/+^ mice (395±18 *vs.* 172±11 MΩ, n = 8/genotype, P<0.001, Fig. 6C). Temporal summation was tested in both genotypes using the same protocol for VB neurons (traces not shown). *Kcne*2 deletion significantly increased summation by 45±3.5% (220±14% vs. 320±16%, n = 8). As was the case with VB neurons, spontaneous EPSPs were evident in both genotypes in the absence (control) of 4-AP; however, the frequency was higher in *Kcne2* ^−/−^ than in *Kcne2* ^+/+^ pyramidal neurons (Fig. 6 A–B). Bath application of 4-AP (0.1 mM) induced rhythmic low threshold Ca^2+^ spike (LTS) burst firing patterns in both genotypes; such bursting could last for more than 90 min. Group data for cortical burst properties are summarized in Fig. 6D. Compared to *Kcne2* ^+/+^, the burst frequency in *Kcne2* ^−/−^ pyramidal neurons was much higher (0.18±0.01 vs. 0.06±0.008 Hz), burst duration was longer (3.4±0.3 s *vs.* 1.2±0.02 s), and the number of fast spikes per burst larger (88.2±7.2 vs. 18.5).

In *Kcne2* ^−/−^ mice, cortical burst duration was also much longer than that seen in VB counterparts (3.4±0.3 s vs. 0.6 s±0.04, P<0.001), a 5.6-fold increase. The magnitude of the effects on voltage responses in *Kcne2* ^−/−^ neurons was much larger in corticothalamic pyramidal neurons than that observed in thalamocortical VB neurons, implying that corticothalamic excitatory transmission was increased in brain slices from *Kcne2* ^−/−^ mice.

### Kcne2 deletion had little effect on glutamate release

**Figure 7:**
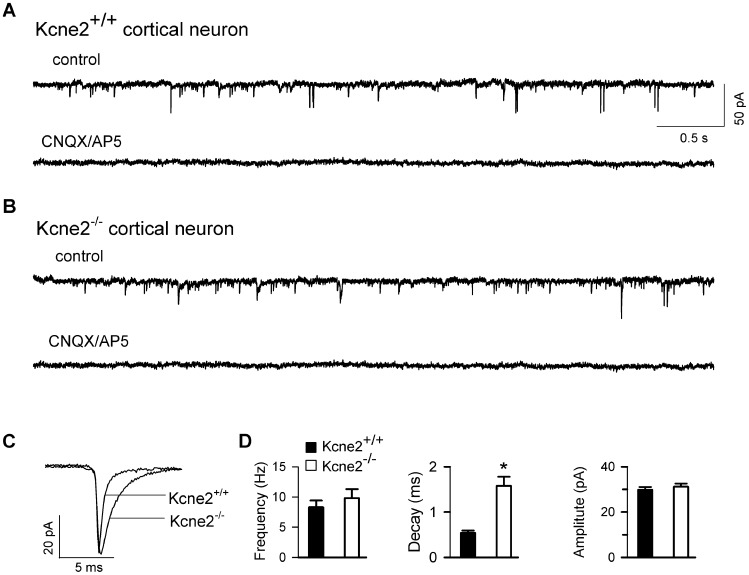
*Kcne2* deletion has little effect on glutamate release. ***A*** and ***B***, Miniature EPSCs (mEPSCs) were recorded in cortical layer 6 pyramidal neurons from both genotypes in the presence of bicuculline (20 µM) and TTX (1 µM) at holding potential of −80 mV. Synaptic currents in both genotypes could be blocked by co-application of CNQX 20 µM and AP5 40 µM. ***C***, Overlay of averaged mEPSCs for the two genotypes. ***D***, Bar graphs comparing mEPSC frequency, decay time and amplitude (n = 5/genotype).

As noted above, the frequency of spontaneous EPSPs in layer 6 pyramidal neurons in the absence of 4-AP was increased by *Kcne2* deletion, suggesting a possible alteration in glutamate release machinery. To investigate this possibility, miniature excitatory postsynaptic currents (mEPSCs) were recorded in both genotypes in the absence of 4-AP but the presence of TTX (500 nM) and bicuculline (20 µM), thereby blocking spike-driven events and fast GABAergic transmission. Fast mEPSCs were readily detected in pyramidal neurons from both genotypes and could be blocked by co-application of CNQX and AP-5 (Fig. 7A–B), confirming that they were mediated by ionotropic glutamate receptors. mEPSC frequency (8.3±1.1 vs. 9.8±1.5 Hz, *Kcne2* ^+/+^ and *Kcne2* ^−/−^, respectively, n = 6 cells/genotype), and amplitude (29.8±1.3 vs. 31.2±1.4 pA) were similar between the two genotypes. mEPSC decay time, however, was significantly prolonged in *Kcne2* ^−/−^ neurons (1.58±0.2 vs. 0.54±0.05 ms) (Fig. 7C–D), and the prolongation in the decay time was consistent with the observed increase in R_in_ (cf. [44]). The data suggest that *Kcne2* deletion does not alter glutamate release from presynaptic glutamatergic neurons, and that the enhanced excitability observed above is likely due to the reduction of shunting produced by I_h_, and appears similar to what has been described for HCN1-null cortical pyramidal neurons [45].

### Kcne2 deletion reduces brain HCN1 and HCN2 protein expression

Co-IP experiments were employed to determine whether KCNE2 forms protein complexes with HCN1 and HCN2 in mouse brain. Western blots using HCN1 or HCN2 antibodies to probe KCNE2 antibody-precipitated fractions did not yield specific signal (data not shown), suggesting either these complexes do not form in mouse brain, that the amount of complex formation is below our detection limit, or our co-IP protocol did not preserve native complexes.

**Figure 8:**
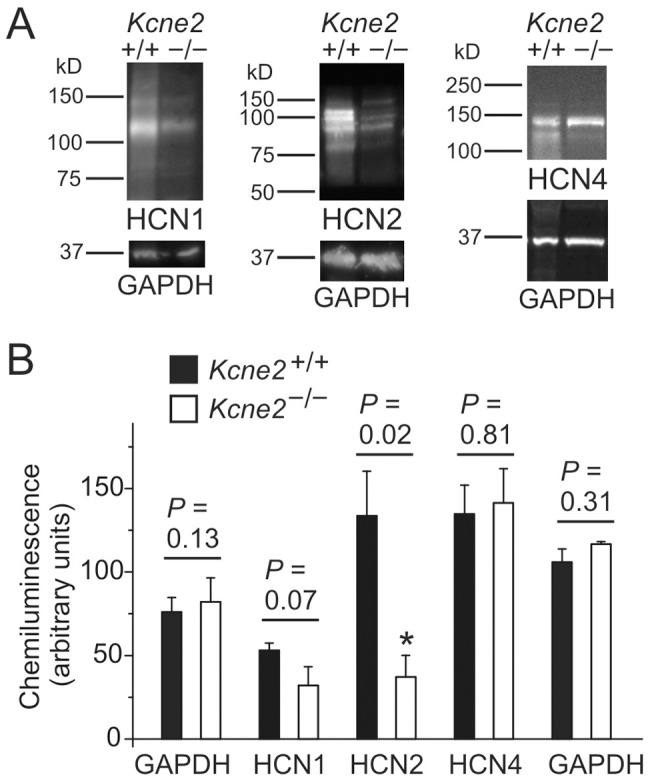
*Kcne2* deletion down-regulates HCN1 and HCN2 protein expression in the brain. ***A***, Exemplar chemiluminescence signals from western blots of whole brain lysates from *Kcne2* ^+/+^ and *Kcne2* ^−/−^ mice, normalized to total protein concentration and probed with antibodies raised against HCN1, HCN2, HCN4 or GAPDH, as indicated. ***B***, Mean chemiluminescence intensities for bands corresponding to known molecular weights for HCN1, HCN2, HCN4 and GAPDH from blots as in panel ***A***, *n* = 3–4 mice per genotype. The cumulative GAPDH data on the left were obtained concomitantly with the HCN1 and HCN2 samples while the comparable GAPDH data on the right were obtained concomitantly with the HCN4 samples. *Significant difference between genotypes at 95% confidence interval. Error bars indicate SEM.

As *Kcne2* deletion reduced native VB and pyramidal neuron I_h_, we also quantified HCN1, HCN2 and HCN4 protein in *Kcne2* ^+/+^ and *Kcne2* ^−/−^ whole-brain lysates to determine if *Kcne2* deletion altered HCN1, HCN2 and HCN4 protein expression. Strikingly, neural expression of HCN2 protein was significantly reduced in *Kcne2*-deleted mice compared to *Kcne2* ^+/+^ mice (*P* = 0.02) and there was a trend toward reduction of HCN1 protein expression (*P* = 0.07) (Fig. 8A–B). This change appears to be specific as HCN4 protein expression was unchanged (Fig. 8A–B) and whole-brain expression of two other membrane proteins, the Kv2.1 K^+^ channel α subunit and the KCC1 K^+^/Cl^−^ co-transporter, was unaffected by *Kcne2* deletion (data not shown).

## Discussion

KCNE2 is a voltage-gated potassium channel ancillary subunit that also regulates heterologously expressed HCN channels. We present novel data which demonstrate that KCNE2 is required for normal HCN channel activity in central neurons.

### Kcne2 deletion impairs neuronal I_h_

Previous studies have provided evidence that the potassium channel β subunit KCNE2 regulates heterologously expressed HCN1, 2 and 4 channels to modulate the activation kinetics and amplitude of I_h_ [11], [14]. Previous investigations of the ability of KCNE2 to modulate HCN gating in heterologous expression systems have yielded variable results. Thus, in *Xenopus* oocytes expressing HCN1 or HCN2, KCNE2 co-expression produces a small (4 mV) depolarizing shift in V_1/2_ [11] while co-expression of KCNE2 with HCN4 produced a significant hyperpolarizing shift (8 mV) in V_1/2_ [12]. In Chinese Hamster Ovary (CHO) cells, KCNE2 co-expression with HCN1, 2, or 4 had no effect on the V_1/2_, although other I_h_ properties were markedly altered [14]. Similarly, over-expression of KCNE2 with HCN2 failed to alter HCN gating in neonatal ventricular myocytes [13]. Given the contradictory results obtained using different expression systems it is therefore necessary, as previously noted [3], to study the effects of native KCNE proteins on native HCN channel function.

### Kcne2 acceleration of current activation

The activation time constants for I_h_ in *Kcne2^+/+^* VB neurons was much slower than that of layer 6 pyramidal neurons (the fast component being 5.6-fold slower and the slow component being 4.5-fold slower), consistent with previous reports of predominant expression of HCN2 in VB and HCN1 in the cortex [16], [17], [41], [42], [46]–[48]. Deletion of *Kcne*2 led to a significant slowing of activation time constants in both VB and cortical neurons (Figs. 2 and 4). Our data are consistent with previous studies using heterologous expression models showing that the presence of KCNE2 accelerates the activation kinetics of all HCN channel subtypes (HCN1, 2 and 4) [13], [14], although there is one report indicating that KCNE2 co-expression with HCN4 slows activation [12].

A comparison of neuronal and recombinant channel properties raises an important question with respect to direct regulation of gating kinetics. When HCN channels are heterologously expressed, the presence of KCNE2 accelerates the kinetics of current activation resulting from homomeric HCN channel expression, and this likely reflects a direct protein-protein interaction [12]. The kinetics of I_h_ in CNS neurons, however, appear to be strongly influenced by HCN channel composition in different cell populations, and expression of heteromultimeric channels may also contribute to differences in the kinetics of neuronal I_h_ [18]. VB neurons primarily express HCN2 channels, with a smaller population of HCN4 channels also present [20], [21]. Given that KCNE2 accelerates I_h_ activation in heterologous expression systems (as discussed above), the slower kinetics of I_h_ observed in *kcne2^−/−^* VB neurons may be due to loss of direct regulation by KCNE2, but could also be due to alterations in HCN subunit expression. As shown in Fig. 8, there is a significant decrease in overall HCN2 expression in the brain when *Kcne2* is knocked out, while overall brain HCN4 expression remains constant. This could theoretically increase the relative contribution of the more slowly activating HCN4 (compared to HCN2) to native I_h_ [48], [49], thereby giving rise to a slowly activating I_h_ current in VB neurons.

KCNE2 protein has been detected in a range of tissues, and its deletion impacts the function of the stomach, heart and thyroid, all of which normally express KCNE2 in mice and humans [10], [19], [31], [32], [50]. While *Kcne2* mRNA has been detected in a range of neural tissues [15], in a recent study we did not observe specific KCNE2 protein staining by immunohistochemistry in neuronal populations of mouse brain. In contrast, robust, highly specific KCNE2 protein staining was apparent in the apical membrane of the choroid plexus epithelium, which lines the fourth and lateral ventricles of the brain and secretes cerebrospinal fluid [19]. It is possible, then, that *Kcne2* deletion alters neuronal I_h_ characteristics indirectly by, for example, altering the cerebrospinal fluid composition in a manner that leads to electrical remodeling such as the reduction in HCN2 protein expression we observed here. This could not only reduce I_h_ density, but also lead to slower-activating I_h_ because of a shift in the balance between HCN2 and HCN4.

### Kcne2 critically regulates excitability and burst firing

Alteration of I_h_ is associated with a marked change in intrinsic excitability in thalamic neurons [33], [34], [43]. Here we found that downregulation of HCN channel function by *Kcne*2 deletion resulted in the increase of input resistance and temporal summation of subthreshold voltage response in both thalamic and cortical neurons, indicating an increase in intrinsic excitability (Figs. 5 and 6). As a result, burst firing also increased in *Kcne2*-null brain slices. Previous studies have shown that genetic downregulation or pharmacological diminishment of the I_h_ conductance is tightly linked to the occurrence of seizure activity [20], [51]–[54] and the decrease of seizure threshold during convulsant challenge [20], [45]. We found that a low concentration of 4-AP induced long-lasting burst firing, which is similar to epileptiform activity [55]–[58]. The burst firing frequency was significantly increased by *Kcne2* deletion in both thalamocortical and layer 6 pyramidal neurons, suggesting increased susceptibility to the convulsant challenge. Intriguingly, the duration of bursts in *Kcne2*-null pyramidal, but not thalamocortical, neurons was much longer, with a marked increase in fast action potentials riding on bursting calcium spikes. Such augmented excitability did not seem to result from a direct alteration of the glutamatergic release machinery as neither mEPSC amplitude nor frequency were changed by the deletion (Fig. 7), but was likely mediated through shunting reduction caused by downregulation of I_h_ (Fig. 6).

HCN1 channels show a 60-fold increase from somatic to distal dendritic membrane in layer 5 pyramidal neurons [37] and not surprisingly, dendritic I_h_ plays a pivotal role in controlling pyramidal cell excitability [59]. A recent study has shown that cortical layer 6 pyramidal dendrites also possess membrane properties similar to those of other layer pyramidal neurons [38]. Hence, downregulation of cortical dendritic I_h_ leads to reduction of dendritic shunting and consequently constraints on local spike propagation to the soma, thereby facilitating synaptic integration and excitatory synaptic transmission. This effect may account for the occurrence of cortical hypersusceptibility to 4-AP observed here, and suggests that *Kcne2*-null mice may be more susceptible to chemical-induced seizures because of dysregulation of HCN channel function. Since 4-AP-induced synchronized oscillations (epileptiform activity) originate in cortical layer 6 and propagate to thalamus through corticothalamic projections [56], the increase in apparent EPSP frequency and burst firing in thalamocortical VB neurons (Fig. 5) may result in part from increased corticothalamic excitatory synaptic input [56], which in turn leads to an enhancement of thalamocortical output to layer 4 and 6 [28], creating an imbalance between excitation and inhibition in the cortico-thalamo-cortical loop. Our results (Fig. 4 and 6) support the notion that dendritic HCN1 plays a critical role for the regulation of cortical pyramidal excitability [45].

KCNE2 can modulate the function of other ion channels [10], [60], including the 4-aminopyridine (4-AP)-sensitive rapidly activated, transient outward potassium current I_A_, which is mediated by the KCND3 gene-product, Kv4.3 [61]. KCNE2 is a β subunit for Kv4.3 [62], and Kv4.3 mRNA is present in the cortex and thalamus [63]; in a thalamic relay neuron model, I_A_ slows the rate of rise and reduces the peak amplitude of the low-threshold Ca^2+^ spike and reducing I_A_ to 0 results in a decrease in the inter-spike interval and in increase in the response of the cell to a depolarizing current injection [64]. Those observations raise the theoretical possibility that the observed changes in excitability observed in thalamic (Fig. 5) and cortical (Fig. 6) neurons resulted from 4-AP block of I_A_.

We do not think that the enhanced excitability in *Kcne2* ^−/−^ mice is the result of changes in Kv4.3 function for the following reasons. Firstly, at membrane potentials less than −65 mV, *Kcne2* deletion by itself does not produce spontaneous spike firing in either thalamic (Fig. 5C, top current trace) or cortical (Fig. 6A, top current trace) neurons, indicating that I_A_-dependent control of spontaneous spike firing is not altered by the deletion. This is not entirely unexpected as I_A_ and I_h_ co-vary and have opposing and complementary effects [65]–[67]. Secondly, there is little if any measurable I_A_ at membrane potentials less than −60 mV, as its V_1/2_ is quite positive (∼−36 mV) [68], and the excitability experiments in the present study were performed at membrane potentials more negative than −65 mV. Thirdly, 4-AP blocks neuronal I_A_ in the low millimolar range, with an IC_50_ of 1 to 2 mM [68], [69] (similar to the IC_50_ of ∼1.5 mM reported in HEK293 cells expressing Kv4.3 channels [70]), and the concentration used to induce burst firing in the present study was 0.1 mM; at this concentration, I_A_ is only blocked by 7% [69]. Finally, absence of I_A_ (as would occur in the presence of full block) produces a very different pattern of activity (at least in a model thalamic cell [64]) than what we observed. In total, these studies strongly indicate that the burst firing patterns observed in the present study did not result from 4-AP modulation of Kv4.3 mediated I_A_ currents.

### Limitations of the present study

Our I_h_ data from VB and pyramidal neurons demonstrate that native HCN gating is KCNE2-dependent inasmuch as *Kcne2* deletion alters native HCN gating, but the lack of native co-immunoprecipitation between KCNE2 and HCN1 or HCN2 from the brain in our study leaves the door open for at least several potential mechanisms for this functional dependence. First, KCNE2 mRNA is clearly detected in cortical and thalamic neurons [15], and it is possible that KCNE2 directly regulates HCN channels in the brain but our biochemical studies did not provide sufficient resolution to detect this, or the necessary conditions to preserve complex stability. Thus, the slowed gating and reduced current density of I_h_ we observe upon *Kcne2* deletion could stem from the loss of KCNE2 from HCN channel complexes, which would be predicted to slow gating and reduce current density *via* reduced single channel conductance [11]–[14]. Second, KCNE2 could function as an ancillary subunit and regulate HCN trafficking to specific regions of the cell membrane in a manner analogous to that of TRIP8B [71]–[74], so in the absence of KCNE2 perhaps HCN surface expression is impaired. Third, it is possible that KCNE2 indirectly impacts neuronal HCN function due to modulatory effects on other ionic currents, including those mediated by a number of voltage-gated potassium channels [10], [60], and that those changes indirectly alter the HCN-mediated current. Fourth, we previously observed that KCNE2 levels were highest in the choroid plexus [19], and KCNE2 is present in many other non-neuronal tissues [50], [75], so the observed effects could be indirect from one or more of a variety of sources within the mouse. These caveats notwithstanding, *Kcne2* deletion is the root cause of the observed effects reported here.

### Conclusion

In summary, voltage-dependent gating of native HCN channels in CNS neurons appears to be controlled by multiple factors, including in some shape or form the β subunit KCNE2, as shown here. The present findings have revealed for the first time that KCNE2 exerts an important role in the maintenance of brain pacemaking function at physiological membrane potentials. Loss of KCNE2 leads to downregulation of HCN channel function associated with increased excitability in neurons in the cortico-thalamo-cortical loop. Thus, KCNE2 strongly influences HCN channel activity crucial for homeostatic regulation of a dynamic balance of excitation and inhibition [76] in the interconnected circuitry. In future work, the specific mechanisms for this functional link will be further investigated.
